# Impact of reduced left ventricular function on repairing acute type A aortic dissection: Outcome and risk factors analysis from a single institutional experience

**DOI:** 10.1097/MD.0000000000012165

**Published:** 2018-08-21

**Authors:** Chun-Yu Lin, Kuang-Tso Lee, Ming-Yang Ni, Chi-Nan Tseng, Hsiu-An Lee, I-Li Su, Heng-Psan Ho, Feng-Chun Tsai

**Affiliations:** a Department of Cardiothoracic and Vascular Surgery; b Department of Cardiology; c Department of Anesthesiology, Chang Gung University, College of Medicine, Chang Gung Memorial Hospital, Linkou, Taiwan.

**Keywords:** cardiopulmonary bypass, heart failure, type A aortic dissection

## Abstract

Preoperative left ventricular dysfunction is a risk factor for postoperative mortality and morbidity in cardiovascular surgeries with cardiopulmonary bypass, including thoracic aortic surgery. Using a retrospective study design, this study aimed to clarify the short- and mid-term outcomes of patients who underwent acute type A aortic dissection (ATAAD) repair with reduced left ventricular function.

Between July 2007 and February 2018, a total of 510 adult patients underwent surgical repair of ATAAD in a single institution. The patients were classified as having left ventricular ejection fraction (LVEF) <50% (low EF group, n = 86, 16.9%) and LVEF ≥50% (normal group, n = 424, 83.1%) according to transesophageal echocardiographic assessment at the operating room. Preoperative demographics, surgical information, and postoperative complication were compared between the two groups. Three-year survival was analyzed using the Kaplan–Meier actuarial method. Serial echocardiographic evaluations were performed at 1, 2, and 3 years postoperation.

Demographics, comorbidities, and surgical procedures were generally homogenous between the 2 groups, except for a lower rate of aortic arch replacement in the low EF group. The averaged LVEFs were 44.3 ± 2.5% and 65.8 ± 6.6% among the low EF and normal groups, respectively. The patients with low EF had higher in-hospital mortality (23.3% versus 13.9%, *P* = .025) compared with the normal group. Multivariate analysis revealed that intraoperative myocardial failure requiring extracorporeal membrane oxygenation support was an in-hospital mortality predictor (odds ratio, 16.99; 95% confidence interval, 1.23–234.32; *P* = .034), as was preoperative serum creatinine >1.5 mg/dL. For patients who survived to discharge, the 3-year cumulative survival rates were 77.8% and 82.1% in the low EF and normal groups, respectively (*P* = .522). The serial echocardiograms revealed no postoperative deterioration of LVEF during the 3-year follow-up.

Even with a more conservative aortic repair procedure, the patients with preoperative left ventricular dysfunction are at higher surgical risk for in-hospital mortality. However, once such patients are able to survive to discharge, the midterm outcome can still be promising.

## 1 Introduction

Repair of acute type A aortic dissection (ATAAD) is a complex and emergent cardiovascular operation that is associated with high postoperative morbidity and mortality. Despite the advancements of diagnostic tools, management algorithms, and surgical and anesthetic technique in the recent era, surgery for ATAAD still represents a challenging field for the cardiothoracic surgeon owing to its complex vascular anatomy and unstable hemodynamics. The in-hospital mortality rates were reported as 18% to 25% in the international registry of acute aortic dissection and 17% in the German registry for acute aortic dissection type A, respectively.^[1,2]^ The patients with impaired left ventricular function who undergo cardiovascular surgeries are with increased risk of postoperative complications, including renal injury, organ malperfusion, myocardial failure, and reduced survival.^[3–6]^ However, results of patients with preoperative left ventricular dysfunction who underwent repair of ATAAD remain to be fully elucidated in previous literature. With a retrospective design, this study aimed to clarify the early and midterm outcomes among patients who underwent repair of ATAAD with reduced left ventricular function based on the individual center experience.

## 2 Patients and methods

### 2.1 Patient enrolment and preoperative management

The present study was conducted with the approval of the Institutional Ethics Committee (no. 201800311B0). The need for informed consent was waived due to the retrospective nature of the study. A total of 510 consecutive patients underwent emergent surgery for ATAAD in a single institution between July 2007 and February 2018. The patients were classified as preoperative left ventricular ejection fraction (LVEF) <50% (low EF group, n = 86, 16.9%) and LVEF ≥50% (normal group, n = 424, 83.1%) according to echocardiographic assessment at the operating room (OR).

The laboratory examinations and image survey were performed in the emergency department (ED). The extent of aortic dissection was diagnosed by experienced radiologist with helical *computed tomography*. Before being transferred to the OR, patients’ hemodynamics were stabilized with intravenous beta-blockage to maintain systolic blood pressure below 120 mm Hg and heart rate below 60 beats per minute according to the 2010 American College of Cardiology/*American Heart Association* guidelines for thoracic aortic disease.^[7]^ Once patients presented with cardiac tamponade in ED, controlled pericardial fluid drainage was performed via *subxiphoid pericardiotomy* or echo-guided pericardiocentesis, and the patients were transferred to the OR to undergo immediate aortic repair.^[8]^ All operations were performed on an emergency basis. European system for cardiac operative risk evaluation score II was used for evaluate the surgical risk.^[9]^

### 2.2 Echocardiographic studies

After *administration of* general anesthesia and endotracheal intubation, arterial pressure catheters were inserted into bilateral radial arteries, and transesophageal echocardiography (TEE) was placed. Prior to sternotomy, the echocardiographic studies were performed using the commercially available echocardiography systems (Vivid 7, GE Healthcare, Milwaukee, WI) by specialized cardiovascular anesthesiologists. Quantitative assessment of left ventricular systolic function *was* proceeded by using the modified Simpson's method in the standard 4- and 2-chamber views during 3 to 5 consecutive cardiac cycles.^[10,11]^ Simultaneously, TEE was used to identify the amount of pericardial effusion, extent of intimal flap, location of primary entry tear, and severity of aortic regurgitation (AR).^[12]^ Before weaned off cardiopulmonary bypass (CPB), the competency of the aortic valve, and cardiac function were reassessed. Right ventricular failure was defined as right ventricular fractional area change ≤ 35% or tricuspid annular plane systolic excursion ≤ 16 mm accompanied with difficulty of weaning from CPB or significant dilatation of the inferior vena cava.^[13]^ Follow-up transthoracic echocardiography was performed at 1, 2, and 3 years postoperatively.

### 2.3 Surgical management

Skin preparation was performed from the upper neck to bilateral thigh. Before 2009, our cannulation strategy for repairing ATAAD tended toward more use of femoral artery cannulation. After 2009, the strategy switched to more utilization of right axillary artery cannulation in combination with femoral artery cannulation and selective antegrade cerebral perfusion (SACP) because of the developing familiarity with this approach. After performing midline sternotomy, the right atrium was cannulated via a single dual-staged venous catheter (Medtronic Inc., Minneapolis, MN). Since then, CPB with deep hypothermia was initiated. The aortic arch and its major branches were carefully dissected and exposed. Once ventricular fibrillation presented, the ascending aorta (AsAo) was cross-clamped, and the left heart was vented through the right superior pulmonary vein. Cardiac arrest was induced by a single dose (25–30 mL/kg) of histidine–tryptophan–ketoglutarate (HTK) solution (Custodiol; Essential Pharmaceuticals, LLC, Newtown, PA) through the coronary orifice at the aortic sinus Valsalva or intermittent retrograde cold-blood cardioplegic solution through the coronary sinus. The dissected AsAo was routinely replaced with Dacron prosthetic graft (Vascutek Gelseal; Terumo Cardiovascular Systems Co, Ann Arbor, MI). If intima tear extended to the aortic root with aneurismal root dilatation or TEE found severe AR which was difficult to be repaired, aortic root replacement was performed with composite Valsalva graft. After proximal anastomosis was completed, systemic extracorporeal circulation was stopped except for the brain, which was perfused by the right axillary artery under deep hypothermic circulatory arrest (18–20^o^C). The SACP flow rate was set around 10 to 15 mL/kg/min and right radial arterial pressure *was maintained* above 50 mm Hg. Furthermore, the AsAo stump was de-clamped following with careful inspection of the opened aortic arch. The aortic arch was trimmed until the entry site was excluded, if possible. The distal anastomosis was performed with an open technique. Among the patients with entry tear located at the distal arch or proximal descending aorta combined with preoperative malperfusion or thoracic aortic aneurismal dilatation, a concomitant frozen elephant trunk procedure was performed with covered stent grafts. Once the distal anastomosis was completed, whole-body perfusion resumed, and systemic rewarming *was initiated*. All aortic anastomoses were reinforced with Teflon felt and tissue glue.

### 2.4 Postoperative care and interventions

After undergoing surgical repair for *ATAAD*, all patients were transferred to a specialized cardiovascular intensive care unit (ICU) for further treatment and observation. At 8 hours postoperation, ventilator weaning protocol *was* started if there *was* no active bleeding, unstable hemodynamics, persistent arrhythmia, or signs of organ malperfusion. The platelet count was maintained above 100,000 mm^3^ to reduce the risk of bleeding. Prothrombin time and activated partial thromboplastin time levels were also closely monitored, and coagulopathy was reversed by plasma transfusion. Early renal replacement therapy was aggressively applied if acute renal failure developed after operation according to Acute Kidney Injury Network criteria.^[14,15]^ Further treatments including survey images, endovascular intervention, and surgical exploration for hemostasis or malperfusion were performed without hesitation whenever indicated.

### 2.5 Statistical analysis

Statistical analyses were performed using SPSS for Windows (version 22.0, SPSS, Chicago, IL). Data are presented as means ± standard deviation for continuous variables and as percentages for categorical data. For all analyses, statistical significance was set at *P* < .05. Univariate analyses were performed using independent *t*-test, Mann–Whitney *U* test, chi-square test, or Fisher's exact test to determine group differences in clinical demographics, surgical information, and postoperative complications. Significant variables in univariate analyses of in-hospital mortality (*P* < .05) were dichotomized based on cutoff values, which were determined in receiver operating characteristic (ROC) curve analyses. These dichotomized risk factors were tested in a prediction model of in-hospital mortality using a multivariate logistic regression analysis, Hosmer–Lemeshow test, and area under ROC curve (AUROC) analysis.^[16]^ The Kaplan–Meier method was used to construct a 3-year cumulative survival, which was compared using the log-rank test.

## 3 Results

### 3.1 Patient demographics

Table 1 shows the clinical demographics, comorbidities, preoperative condition, and clinical presentation for the low EF and normal groups. The averaged LVEFs were 44.3 ± 2.5% and 65.8 ± 6.6% in the low EF and normal groups, respectively. A total of 28.6% of patients were female, and the mean age was 55.7 ± 14.1 years. Hypertension was the most prevalent comorbidity accounting for >70% of cases in both groups. The average time interval from ED to OR was 5.5 ± 1.7 hours. Around 19% of the patients were diagnosed with intramural hemorrhage rather than typical aortic dissection. Intractable pain was the most clinical presentation (69.6%), followed by hemopericardium (30.8%) and AR with heart failure symptoms (15.1%). No disparity in clinical presentation was found between the 2 groups.

**Table 1:**
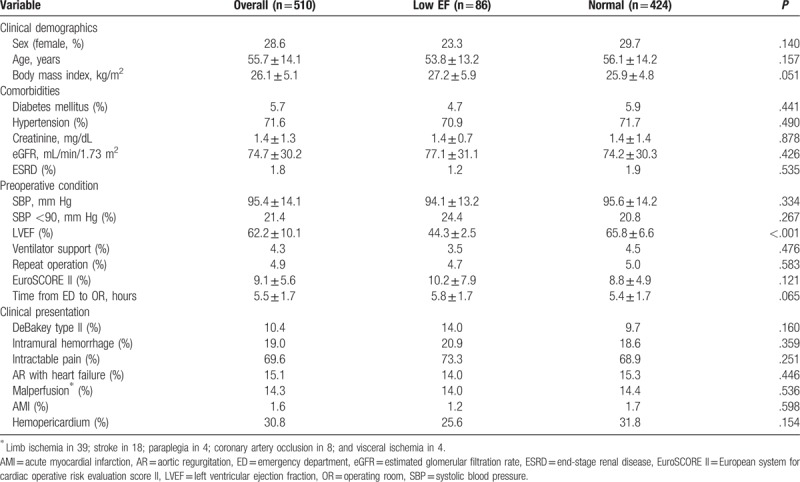
Preoperative characteristics for the low EF and normal groups.

### 3.2 Surgical information

Table 2 provides detailed information regarding surgical variables. The cannulation strategy and prevalence of using SACP were not statistically different between the 2 groups. The aortic repair procedures revealed more aortic arch replacement in the normal group than in the low EF group (26.9% vs 17.4%, *P* = .041). The time span of CPB, aortic cross-clamp, and circulatory arrest revealed no differences between the 2 groups. Moreover, the body temperature and mean arterial pressure during circulatory arrest were also similar. About 11.8% of the patients required Kerlix packing due to coagulopathy with uncontrolled bleeding, and 3.3% underwent extracorporeal membrane oxygenation (ECMO) installation at OR due to intraoperative myocardial failure.

**Table 2:**
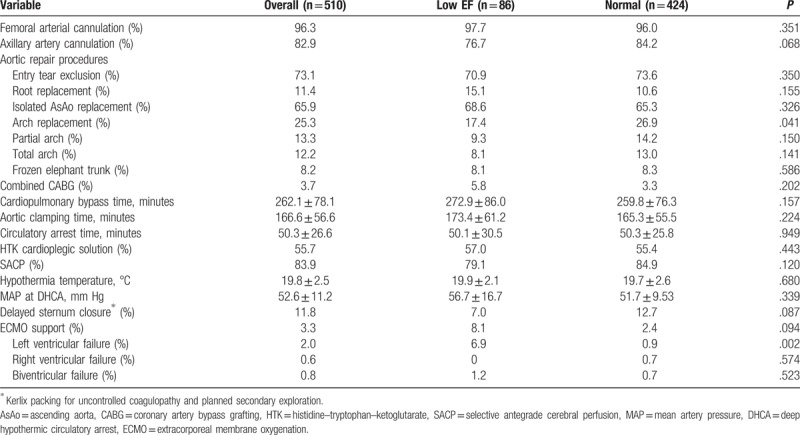
Surgical information for the low EF and normal groups.

### 3.3 Postoperative complications

As shown in Table 3, patients with low EF had significantly higher in-hospital mortality rates compared to patients with normal heart function (23.3% versus 13.9%, *P* = .025). In addition, the low EF group showed a high incidence of complications, including visceral ischemia, limb ischemia, malperfusion-related complication, and ICU readmission, but no statistical significance was observed. Subgroup analyses of outcome among high risk populations are illustrated in Table 4, which revealed that patients with low EF have significant higher in-hospital mortality compared to the normal group if they presented with shock status prior to operation. As for the patients with age of >70 years and complicated with postoperative malperfusion, the low EF group also showed a trend of higher in-hospital mortality.

**Table 3:**
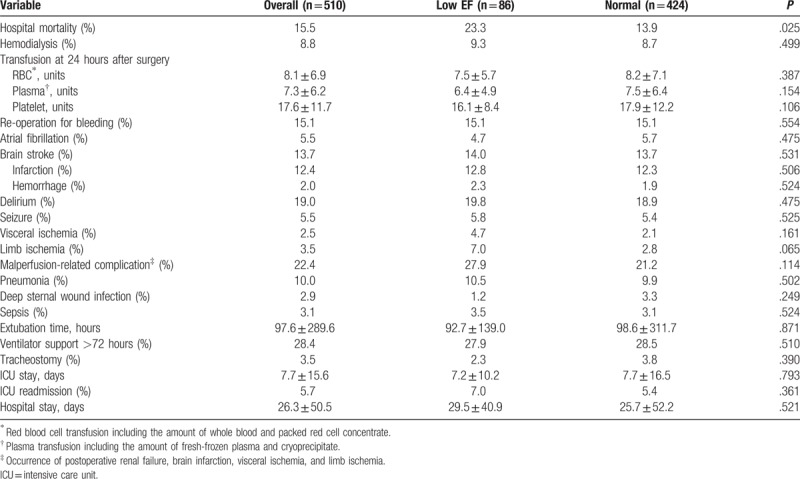
Postoperative mortality and morbidity for the low EF and normal groups.

**Table 4:**
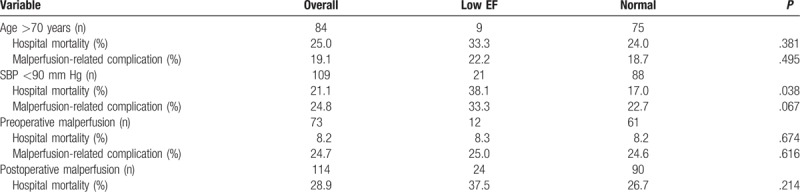
Subgroup analyses of outcome among high-risk populations.

### 3.4 Regression analysis of in-hospital mortality

Table 5 shows the results for the regression analyses among patients with low EF, including those for preoperative serum creatinine >1.5 mg/dL, CPB time >220 minutes, aortic clamping time >170 minutes, ECMO support in the OR, re-exploration for bleeding, and postoperative visceral ischemia. Two significant prognostic factors for in-hospital mortality were identified: ECMO support at OR (odds ratio (OR), 16.99; 95% confidence interval (CI), 1.23–234.32; *P* = .034) and preoperative serum creatinine >1.5 mg/dL (OR 7.03; 95% CI, 1.90–25.78; *P* = .003). For serum creatinine, the established model revealed a good calibration (Hosmer–Lemeshow test, *P* = .52) and discrimination (AUROC 0.711, *P* = .004).^[16]^

**Table 5:**
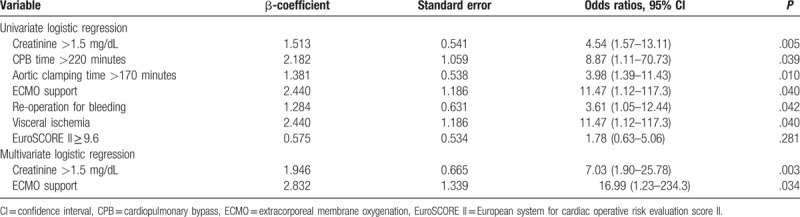
Logistic regression analysis for hospital mortality of 86 patients in the low EF group.

### 3.5 Cumulative survival and left ventricular function at 3 years

**Figure 1:**
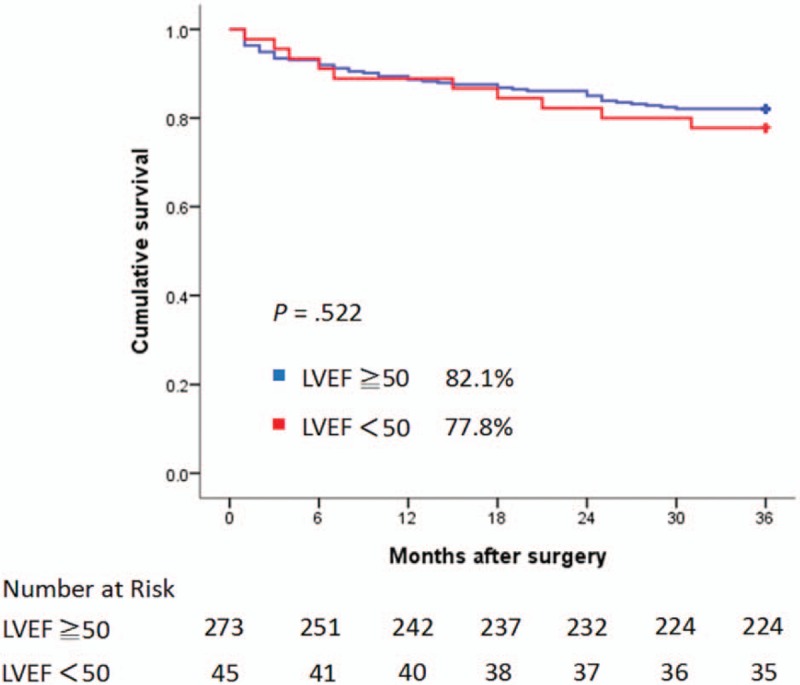
Kaplan–Meier curves for cumulative survival stratified by left ventricular ejection fraction.

**Figure 2:**
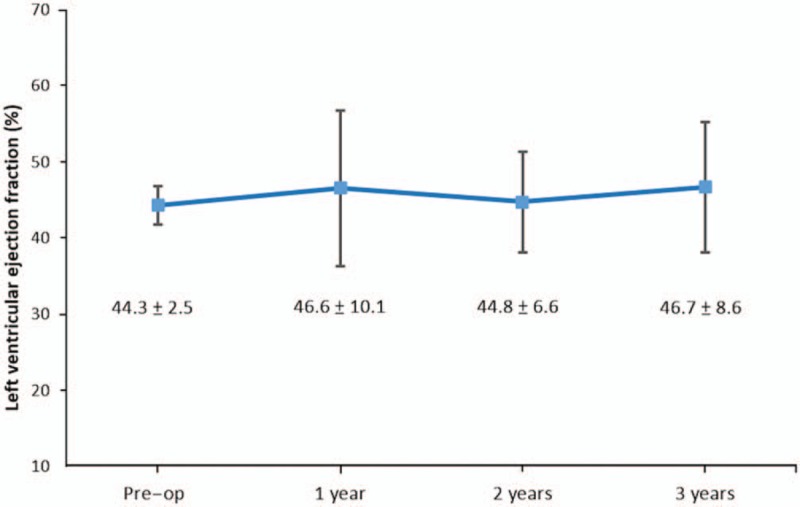
Echocardiographic measurements of left ventricular performance preoperatively and in 3 years post repair of acute type A aortic dissection.

As Figure 1 illustrated, for patients who survived to discharge, the 3-year cumulative survival curves were not significantly different between the low EF and normal groups (77.8% vs 82.1%, *P* = .522). Furthermore, the serial echocardiograms revealed no deterioration of LVEF at 3-year follow-up compared to the preoperative status (Fig. 2).

## 4 Discussion

Low preoperative LVEF is common in patients undergoing cardiovascular surgery, especially those scheduled for coronary artery bypass graft surgery and valve surgery.^[17]^ and accounted for 15% to 36% of patients who underwent repair of ATAAD.^[17,18]^ Some previous literatures also demonstrated that it can jeopardize the survival and increase complications of aortic surgery.^[19–21]^ In this single-center study, a comparatively large cohort of patients who underwent emergency surgery for ATAAD was presented, including 86 patients with reduced left ventricular function. In-hospital mortality of this population is significantly higher than those with normal heart function. However, once such patients are able to survive to discharge, the midterm outcome can still be promising.

### 4.1 Intraoperative myocardial failure and mechanical support

Left ventricular dysfunction occurs due to loss of functional myocytes or a decrease in their function. CPB with cardioplegic arrest can lead to myocardial dysfunction, which results from ischemic/reperfusion injury of the heart. The persistence of such dysfunction may vary from temporary stunning to persistent myocardial infarction and failure.^[21]^ As Hamad et al^[3]^ reported, patients with preoperative left ventricular systolic dysfunction could develop low cardiac output syndrome (LCOS) more frequently than do patients with a normal LVEF. Furthermore, it is well established in the cardiothoracic surgical literature that extended CPB and aortic cross-clamping time are significant risk factors for mortality and morbidity in patients undergoing cardiac surgery.^[23,24]^ In the present study, we found that the time spans of CPB and aortic cross-clamp are both long which may be associated with the complexity of ATAAD surgery. Unsurprisingly, the patients with pre-existing impaired LVEF would exhibit high possibility of intraoperative myocardial failure and require mechanical support. Placement of intra-aortic balloon pump in patients who develop myocardial failure after cardiac surgery may be beneficial to survival. However, using intra-aortic balloon in patients with acute aortic dissection is not a safe modality owing to the risk of making the dissected aorta more injurious and even ruptured. Therefore, once patients presented with intraoperative myocardial failure refractory to inotropic medication, ECMO installation was performed at OR. In a previous study reported from this institute, the application of ECMO for stabilizing intraoperative myocardial failure was introduced since 2003.^[25]^ As reported by Lin et al,^[26]^ postoperative ECMO requirement predicted an elevated risk of in-hospital death among ATAAD, and it was more frequently required in the presence of preoperative shock or increased aortic cross-clamp time. In the present study, ECMO support at OR was also a significant independent predictor of in-hospital mortality for the low EF group. Furthermore, even without requiring a mechanical support, these patients’ hemodynamics may be maintained with high-dose inotropes, which potentially induce complications such as arrhythmia, organ failure, and infection.

### 4.2 Perioperative TEE assessment

ATAAD is a life-threatening cardiovascular emergency. Therefore, prompt surgical repair to prevent rupture and relative complications is critical to the outcome. In the present study, the prevalence of hypertension is >70% among patients who underwent ATAAD repair. Hypertension is highly associated with atherosclerotic cardiovascular disease, which could increase surgical risk. Preoperative cardiac evaluation is important to allow identification of those patients at higher risk for a cardiac event after CPB. However, arranging a regular preoperative examination, which would delay the surgical timing, is commonly difficult. Therefore, a quick and efficient cardiac assessment by perioperative TEE can provide valuable information for surgeons to make surgical planning according to different risk populations.^[27]^ As an important diagnostic tool in cardiac surgery, it can be used to reveal the etiology of LCOS and assess heart volumes, systolic and diastolic function, valve pathology, pulmonary circulation, ventricular filling pressures, pericardial effusion, and fluid responsiveness.^[22]^ In our institute, the ATAAD repair strategy could be toward conservative if reduced cardiac function is detected by TEE before initiating CPB. However, the baseline data of cardiac function prior to ATAAD was not available. Therefore, we were unable to compare the changes in cardiac function before and after this cardiovascular event. It was also difficult to establish definite causality about whether the reduced cardiac function was pre-existing or caused by ATAAD. In general, a clinical diagnosis was made according to the symptoms, preoperative studies, and experienced echocardiographic assessment. If significant aortic valve regurgitation or compromised coronary ostia were identified by TEE, accompanied by dyspnea, myocardial ischemia on electrocardiogram, elevated cardiac enzyme, or pulmonary edema on *computed tomography, the reduced cardiac function may be secondary to ATAAD.* Otherwise, a pre-existing etiology is more likely to be suspected.

### 4.3 Preoperative renal dysfunction

In the present study, preoperative renal dysfunction was also identified as an independent predictor of in-hospital mortality for the low EF group. A similar result has been described in the study of Wang et al.^[28]^ The overall incidence of renal dysfunction and renal replacement therapy after thoracic aortic surgery has been reported to be high compared with other cardiac operations.^[29]^ Moreover, postoperative renal replacement therapy could increase the in-hospital mortality rates in patients who underwent emergent thoracic aortic surgery, including ATAAD.^[30]^ The patients without preoperative renal dysfunction may endure acute kidney injury with less progression to acute renal failure because of better-preserved preoperative renal function. In other words, preoperative renal dysfunction would elevate the risk of postoperative acute renal failure if acute kidney injury occurred. Furthermore, the patients with low EF had a high prevalence of chronic hypertension and preoperative shock status, which may both lead to impaired autoregulation of kidney function.^[31]^

### 4.4 Strategy of myocardial protection

In the present study, HTK solution was used as the myocardial protective agent in >50% of patients who underwent repair of ATAAD. According to studies by Scrascia and Perera,^[32,33]^ HTK and cold-blood cardioplegic solutions assure similar myocardial protection in patients undergoing thoracic aorta operations. However, both studies did not consist of pure ATAAD populations (27%–40%). The patients with ATAAD have a high prevalence of chronic hypertension, which is usually associated with left ventricular hypertrophy and may increase the demanding dosage of cardioplegic solution. In addition, as a life-threatening emergency, preoperative evaluation of coronary artery disease is usually inadequate. Therefore, further studies on myocardial protection strategies in pure ATAAD population should be conducted to clarify this important issue.

### 4.5 Limitations of this study

Despite the promising results of the present study, several important limitations must be considered. First, because the study used a retrospective and nonrandomized control design, bias might exist influencing the homogeneity of the study and controlled groups. Furthermore, causality cannot be established from this retrospective review. Other confounding factors that were not observed might have caused the early mortality difference. Second, the decision regarding repair procedures was made by individual physicians. A different extent for aortic replacement and strategies of secondary intervention might have also affected the final outcomes. Third, as an 11-year crossed cohort, technology of CPB, strategy of myocardial protection, and ICU care protocol may change in different eras. Finally, as a retrospective study, some hemodynamic data, laboratory profiles, and inotropic medication dosage information were not completely analyzed due to incomplete records. This hindered more detailed analyses of physiological fluctuations during perioperative course.

## 5 Conclusions

Left ventricular dysfunction is a minority group in the ATAAD surgical population but is associated with high in-hospital mortality and morbidity. The patients with reduced cardiac function and who required complex aortic repair procedures with prolonged CPB are expected to have high possibility of early mortality. However, once such patients are stabilized by surgical treatment and survive to discharge, the midterm outcome can still be promising compared to patients in the normal group. The accurate planning of surgical management and identification of high-risk patients combined with aggressive myocardial supports using pharmacological and mechanical measures are mandatory to improve clinical outcomes.

## Acknowledgments

We thank Tao-Hsin Tung, Department of Medical Research and Education, Cheng-Hsin General Hospital, for statistical assistance.

## Author contributions

**Conceptualization:** Chun-Yu Lin, Ming-Yang Ni, Feng-Chun Tsai.

**Data curation:** Chun-Yu Lin, I-Li Su, Heng-Psan Ho.

**Investigation:** Chun-Yu Lin.

**Methodology:** Chun-Yu Lin, Kuang-Tso Lee, Chi-Nan Tseng, Hsiu-An Lee, Feng-Chun Tsai.

**Project administration:** Chun-Yu Lin.

**Resources:** Chun-Yu Lin.

**Supervision:** Chun-Yu Lin.

**Writing – original draft:** Chun-Yu Lin.

**Writing – review & editing:** Chun-Yu Lin, Kuang-Tso Lee, Ming-Yang Ni, Chi-Nan Tseng.
