# Impact of reduced left ventricular function on repairing acute type A aortic dissection: Outcome and risk factors analysis from a single institutional experience: Erratum

**DOI:** 10.1097/MD.0000000000012571

**Published:** 2018-09-21

In the article, “Impact of reduced left ventricular function on repairing acute type A aortic dissection: Outcome and risk factors analysis from a single institutional experience”,^[1]^ which appeared in Volume 97, Issue 35 of *Medicine*, Table 5 needs to be corrected so that EuroSCORE II≥9.6 appears as EuroSCORE II > 9.6.

**Table 5:**
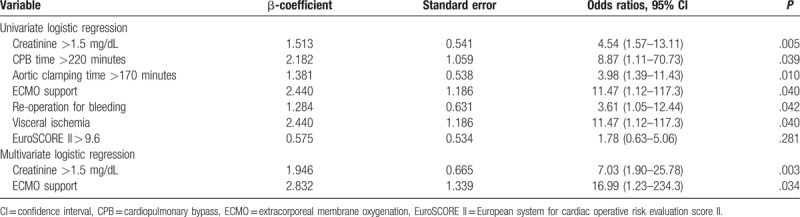
Logistic regression analysis for hospital mortality of 86 patients in the low EF group.
